# Etiologic Patterns and Evolution of Healthcare-Associated Infections in the Pandemic and Post-Pandemic Periods: A County-Level Multicenter Study from Southeastern Romania

**DOI:** 10.3390/antibiotics15020214

**Published:** 2026-02-15

**Authors:** Corina Voinea, Elena Mocanu, Elena Dantes, Sanda Jurja, Ana-Maria Neculai, Aurora Craciun, Lucian Serbanescu, Ana-Maria Dascalu, Mihaela Cezarina Mehedinti, Sorin Rugina

**Affiliations:** 1 Doctoral School of Medicine, Faculty of Medicine, Ovidius University of Constanta, 1 University Alley, Campus—Corp B, 900470 Constanta, Romania; corina.badescu2012@gmail.com (C.V.); elena.dantes@365.univ-ovidius.ro (E.D.); sanda.jurja@365.univ-ovidius.ro (S.J.); sorinrugina@yahoo.com (S.R.); 2 Public Health Directorate Constanta, 1 Lacramioraei Alley, 900643 Constanta, Romania; 3 Department of Public Health and Management, Faculty of Medicine, Ovidius University of Constanta, 1 University Alley, Campus—Corp B, 900470 Constanta, Romania; 4 Department of Pneumology, Faculty of Medicine, Ovidius University of Constanta, 1 University Alley, Campus—Corp B, 900470 Constanta, Romania; 5 Clinical Hospital of Pneumopthisiology Constanta, 40 Santinelei Street, 900002 Constanta, Romania; 6 Department of Ophthalmology, Faculty of Medicine, Ovidius University of Constanta, 1 University Alley, Campus—Corp B, 900470 Constanta, Romania; 7 County Clinical Emergency Hospital “St. Apostle Andrew” Constanța, 145 Tomis Boulevard, 900591 Constanta, Romania; lucian.serbanescu@365.univ-ovidius.ro; 8 Department of Biochemistry, Faculty of Medicine, Ovidius University of Constanta, 1 University Alley, Campus—Corp B, 900470 Constanta, Romania; anamneculai89@gmail.com (A.-M.N.); aurora.craciun@365.univ-ovidius.ro (A.C.); 9 Department of Obstetrics—Gynecology, Faculty of Medicine, Ovidius University of Constanta, 900470 Constanta, Romania; 10 Department of Ophthalmology, Faculty of Medicine, University of Medicine and Pharmacy “Carol Davila”, 020021 Bucharest, Romania; ana.dascalu@umfcd.ro; 11 Emergency University Hospital Bucharest, Splaiul Independenței nr. 169, Sector 5, 050098 Bucharest, Romania; 12 Department of Functional and Morphological Science, Faculty of Medicine and Pharmacy, Dunarea de Jos University, 800010 Galati, Romania; mihaela.mehedinti@ugal.ro; 13 Romanian Academy of Medical Sciences, 1 I.C. Brătianu Boulevard, Sector 3, 030171 Bucharest, Romania; 14 Academy of Romanian Scientists, Ilfov Street, No. 3, Sector 3, 050044 Bucharest, Romania

**Keywords:** healthcare-associated infections, *Clostridioides difficile*, COVID-19, hospital epidemiology, post-pandemic

## Abstract

**Background/Objectives**: Healthcare-associated infections (HAIs) remain a major source of morbidity, mortality, and healthcare burden, and were profoundly affected by the COVID-19 pandemic through changes in case mix, care organization, and antimicrobial use. This study aimed to compare the epidemiology, etiology, ward distribution, risk factors, and outcomes of HAIs during the pandemic and post-pandemic periods in southeastern Romania, with particular emphasis on Clostridioides difficile infection (CDI), multidrug-resistant (MDR) pathogens, and in-hospital mortality. **Methods**: This retrospective observational study included 3929 patients with confirmed HAIs reported by 10 hospitals in one Romanian county between March 2020 and December 2024, divided into a pandemic period (March 2020–March 2022) and a post-pandemic period (April 2022–December 2024). Sociodemographic, clinical, ward-related, therapeutic, and microbiological variables, together with discharge status and cause of death, were analyzed using Fisher’s exact test, Z-tests with Bonferroni correction, the Mann–Whitney U test, and multivariable models, applying national and ECDC-aligned surveillance definitions for HAIs. **Results**: Patients were predominantly older adults (median age 67 years), with a slight male and urban predominance. Hospital stays were longer during the pandemic. Immunosuppression, previous surgery, antisecretory therapy, and chemotherapy were more frequent post-pandemic. HAIs were mainly reported from medical wards, with a relative shift towards intensive care units during the pandemic; pediatric wards carried a smaller burden. CDI was the leading HAI (about half of all cases) with higher post-pandemic prevalence, whereas SARS-CoV-2 infections predominated in medical and surgical wards; *Acinetobacter baumannii* and *Klebsiella pneumoniae* clustered in intensive care units during the pandemic, and were more often associated with mortality. Overall, 59.7% of patients improved and 17.5% died, with higher mortality during the pandemic, while post-pandemic deaths were more frequently unrelated to HAIs. **Conclusions**: This study demonstrates a substantial and ongoing burden of healthcare-associated infections in southeastern Romania, with elderly patients with prolonged hospital stays and complex medical conditions being most affected and experiencing considerable mortality, particularly in medical and intensive care units. After the pandemic, *Clostridioides difficile* infections became more prevalent in the context of repeated antibiotic use and immunosuppression. Mortality among patients with HAIs was higher during the pandemic, whereas in the post-pandemic period deaths were more often unrelated to HAIs, underscoring the need to strengthen antimicrobial stewardship programs and infection prevention strategies.

## 1. Introduction

Healthcare-associated infections (HAIs) remain a major global public health issue, significantly increasing morbidity, mortality, and healthcare costs. The COVID-19 pandemic exacerbated this problem by disrupting infection prevention and control (IPC) programs and promoting extensive antibiotic use, which accelerated the spread of multidrug-resistant (MDR) pathogens [1]. *Acinetobacter baumannii*, *Klebsiella pneumoniae*, *Pseudomonas aeruginosa*, and *Staphylococcus aureus* are frequently identified as predominant pathogens in serious nosocomial infections among critically ill COVID-19 patients [2]. As the pandemic subsides, understanding post-pandemic healthcare-associated infection surveillance is essential for guiding future infection prevention and control measures and improving antimicrobial stewardship in hospitals.

This study examines how the COVID-19 pandemic affected the incidence, etiology, and microbiological profile of healthcare-associated infections reported by hospitals in a southeastern Romanian county. The analysis contrasts two periods: the pandemic phase (March 2020–March 2022) and the post-pandemic phase (April 2022–December 2024). Key variables include demographic characteristics (age, sex, place of residence), clinical parameters (hospital ward type, length of stay, comorbidities, immunosuppressive status), therapeutic history (previous hospitalizations, antimicrobial exposure, surgical interventions), and microbiological data (infection site, isolated pathogens). While previous studies have shown increased secondary infections and concern about antibiotic resistance in COVID-19 patients globally [3], there is less understanding of the epidemiological progression of healthcare-associated infections (HAIs) after the pandemic. Romania also has a distinctive healthcare environment with varied IPC resources and evolving diagnostic capabilities, highlighting the need for locally derived evidence [4].

This study is necessary due to the deficit of longitudinal data on HAIs beyond the acute pandemic phase. During the COVID-19 crisis, the strain on intensive care units (ICUs) and workforce shortages led to reduced compliance with infection prevention and control (IPC) regulations and excessive use of broad-spectrum antibiotics, promoting resistance [5]. Although emergency measures have been relaxed and infection control surveillance can resume, problems remain, such as inconsistent case reporting and the continued presence of MDR strains in hospitals [6]. Therefore, it is essential to examine how pandemic-related disruptions have altered HAI patterns and the factors influencing mortality in Romanian healthcare facilities.

Although many international studies document increased HAI incidence and mortality during the pandemic [7], comparative analyses between the pandemic and post-pandemic periods are largely absent from Romanian research. Under-reporting and inconsistencies in national surveillance systems also hinder accurate epidemiological assessments [8]. This study aims to answer the following questions: what were the epidemiological, etiological, and clinical developments of healthcare-associated infections during the pandemic and post-pandemic periods in southeastern Romania, and what are the risk factors and prognostic determinants of these infections?

This research offers both theoretical and practical contributions. Theoretically, it provides a comprehensive longitudinal analysis of HAI trends over five years, encompassing both pandemic disruptions and post-epidemic stabilization. In practice, the results are intended to support Romania’s national IPC and antimicrobial stewardship policies and help the country meet European surveillance standards. Ultimately, this research aims to improve understanding of post-pandemic infection dynamics and guide targeted measures to reduce the burden of HAIs in the Romanian healthcare system by incorporating local epidemiological data into a broader European context.

## 2. Results

### 2.1. Demographic and Clinical Characteristics of the Patients

The study included 3929 patients with confirmed HAIs reported by 10 hospitals in one Romanian region, of whom 1754 were hospitalized during pandemic period and 2175 during post-pandemic period. The demographic and clinical characteristics of the patients are presented in Table 1. The median age of the patients was 67 years (IQR: 55–76 years). Sex distribution showed a predominance of male patients (52.2%), with a significantly higher proportion observed during the pandemic period compared with the post-pandemic period (54.8% vs. 50.1%, *p* = 0.004). Most patients were from urban areas (69.4%), with this background more frequently observed during the pandemic period than in the post-pandemic period (71.2% vs. 69.4%, *p* = 0.028).

The median length of hospital stay was 15 days (IQR: 10–22), significantly longer during the pandemic period (16 days, IQR: 10–24) compared with the post-pandemic period (14 days, IQR: 9–21; *p* < 0.001).

Immunosuppression was present in 13.6% of patients and was significantly more frequent in the post-pandemic period compared with the pandemic period (21.8% vs. 3.4%; *p* < 0.001). Previous surgical interventions were reported in 8.8% of patients, with a higher frequency in the post-pandemic period (12.7% vs. 3.9%; *p* < 0.001).

### 2.2. Type of Hospital Ward and Period of Hospitalization

Most patients were admitted to medical wards (77.9%). Admissions to intensive care units were significantly more frequent during the pandemic period (6.0% vs. 2.6%), whereas admissions to medical wards predominated in the post-pandemic period (79.2% vs. 76.3%; *p* < 0.001) (Table 2).

### 2.3. Types of Healthcare-Associated Infections

**Figure 1:**
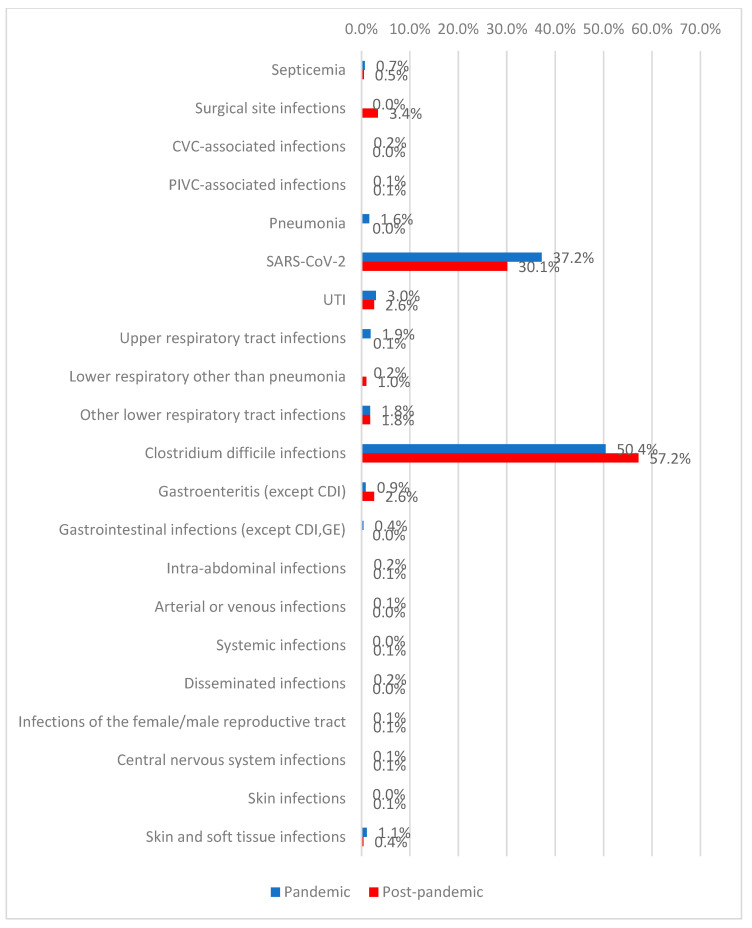
Distribution of patients according to HAI type and period of admission.

The most frequently identified healthcare-associated infection was *Clostridioides difficile* infection (54.2%), followed by SARS-CoV-2 infection (33.2%). During the post-pandemic period, higher frequencies were observed for surgical site infections (3.4% vs. 0%), non-pneumonic lower respiratory tract infections (1.0% vs. 0.2%), *C. difficile* infections (57.2% vs. 50.4%), and gastroenteritis (2.6% vs. 0.9%). In contrast, pneumonia (1.6% vs. 0%), upper respiratory tract infections (1.9% vs. 0.1%), gastrointestinal infections (0.4% vs. 0%), and skin and soft tissue infections (1.1% vs. 0.4%) were more frequently diagnosed during the pandemic period (*p* < 0.001) (Figure 1, Table 2).

### 2.4. Etiology of Healthcare-Associated Infections

**Figure 2:**
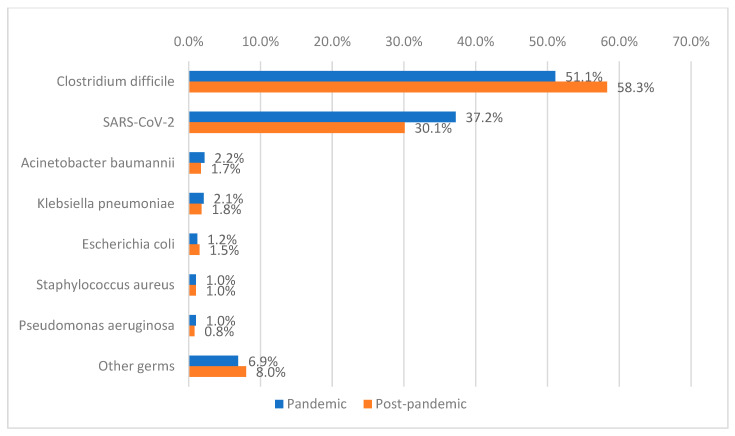
Distribution of patients according to etiology and period of admission.

Among the patients included in the study, 2.9% had healthcare-associated infections with multiple etiologies, while 97.1% had a single identified etiological agent. *Clostridioides difficile* was the predominant etiological agent (55.1%), with a significantly higher prevalence in the post-pandemic period compared with the pandemic period (58.3% vs. 51.1%; *p* < 0.001). SARS-CoV-2 was detected in 33.2% of patients. Other isolated pathogens included *Acinetobacter baumannii* (2.0%), *Klebsiella pneumoniae* (1.9%), *Escherichia coli* (1.3%), *Staphylococcus aureus* (1.0%), *Pseudomonas aeruginosa* (0.9%), and other bacteria (7.5%), the latter being significantly more frequent during the pandemic period (6.9% vs. 8%; *p* = 0.182) (Figure 2, Table 2).

### 2.5. Medical History and Prior Exposures Among CDI Patients

**Figure 3:**
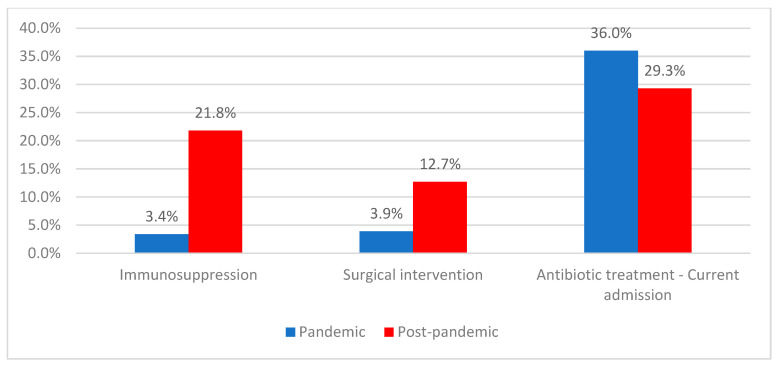
Distribution of patients according to the presence of immunosuppression, surgical intervention, antibiotic treatment at current admission and period of admission.

Among patients with *Clostridioides difficile* infection, 58.7% had been hospitalized within the previous year, most commonly less than four weeks before the current admission (75.4%). Antibiotic treatment at the time of the current admission was given to 32.3% of patients and was more frequent during the pandemic period (36.0% vs. 29.3%; *p* < 0.001) (Table 3, Figure 3).

**Figure 4:**
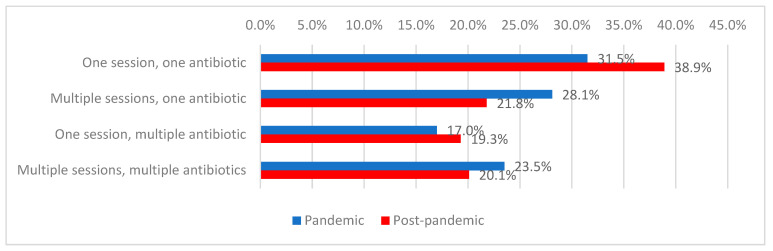
Distribution of patients according to the type of antibiotic treatment at their current admission and period of admission.

The use of a single antibiotic regimen consisting of one antimicrobial agent was the most common approach (35.2%), predominantly observed in the post-pandemic period (38.9% vs. 31.5%). In contrast, multiple regimens involving a single antibiotic were more frequently used during the pandemic period (28.1% vs. 21.8%; *p* = 0.006) (Table 3, Figure 4).

**Figure 5:**
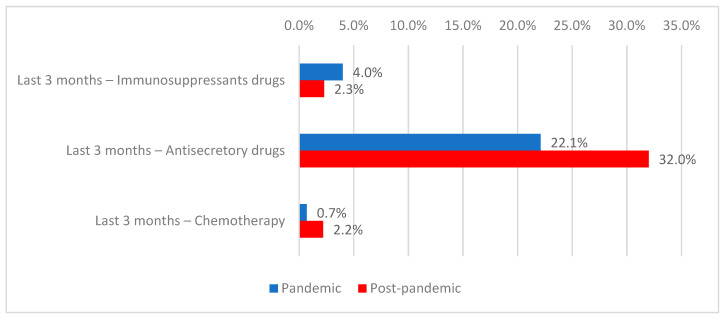
Distribution of patients according to treatment in the last three months and period of admission.

In the previous three months, 16.1% of patients had received antibiotics, 3.0% immunosuppressive agents (more frequently during the pandemic period: 4.0% vs. 2.3%; *p* = 0.002), 27.6% antisecretory medications (more frequently in the post-pandemic period: 32.0% vs. 22.1%; *p* < 0.001), and 1.5% chemotherapy (more frequently in the post-pandemic period: 2.2% vs. 0.7%; *p* < 0.001) (Table 3, Figure 5).

**Figure 6:**
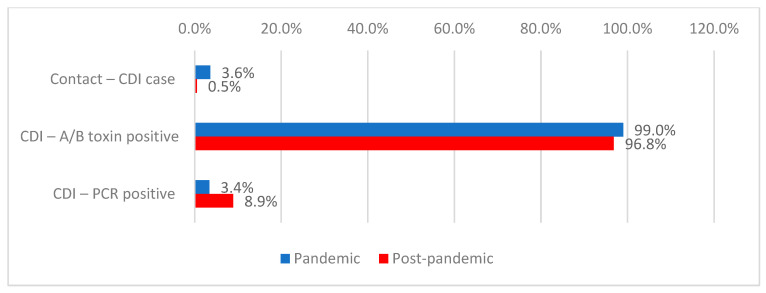
Distribution of patients with *Clostridioides difficile* infection according to the presence of CDI contact, detection and period of admission.

Among patients with *Clostridioides difficile* infection, prior contact with positive cases was reported in 1.8% of cases, occurring more frequently during the pandemic period (3.6% vs. 0.5%; *p* < 0.001). Toxins A/B were detected in 97.7% of patients, with a higher frequency during the pandemic period (99.0% vs. 96.8%; *p* = 0.001), while PCR testing was positive in 6.6% of cases, more frequently during the post-pandemic period (8.9% vs. 3.4%; *p* < 0.001). Pseudomembranous colitis on colonoscopy was documented in 0.2% of cases, and histopathological confirmation in 0.1% (Table 3, Figure 6).

### 2.6. Discharge Status

**Figure 7:**
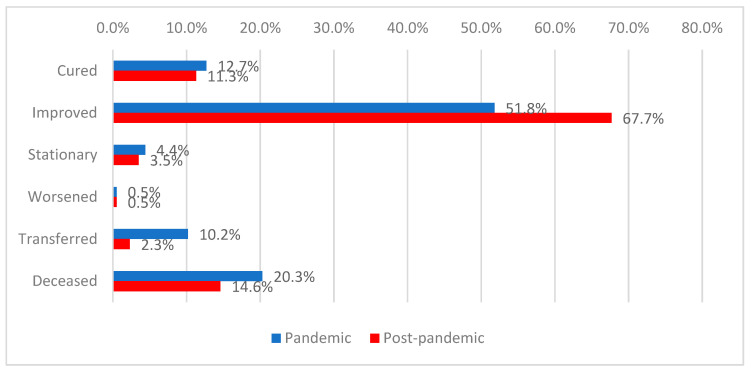
Distribution of patients according to discharge status and period of admission.

The majority of patients had a favorable outcome at discharge (59.7%), while 17.5% of the analyzed patients died. A favorable outcome was significantly more frequent among patients hospitalized during the post-pandemic period (67.7% vs. 51.8%), whereas transfers (10.2% vs. 2.3%) and deaths (20.3% vs. 14.6%) were more common during the pandemic period (*p* < 0.001) (Figure 7).

**Figure 8:**
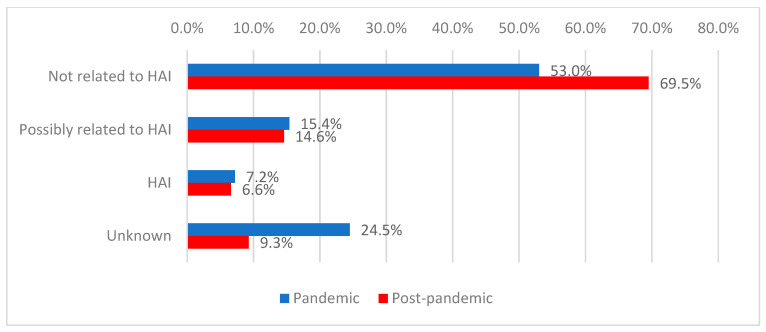
Distribution of deceased patients according to cause of death and period of admission.

Among deceased patients, in most cases the cause of death was not attributed to healthcare-associated infections (59.8%), a situation more frequently observed during the post-pandemic period (69.5% vs. 53.0%). Deaths with an unknown cause were significantly more frequent during the pandemic period (24.5% vs. 9.3%; *p* < 0.001) (Figure 8).

### 2.7. Distribution of Patients by Ward Type and Etiology

The analysis of patient distribution by ward type and etiology (Table 4) revealed significant differences in both the overall cohort and between the two analyzed periods.

Overall, *Clostridioides difficile* infections were more frequent in the Emergency Department, whereas SARS-CoV-2 infections predominated in medical and surgical wards. Infections caused by *Acinetobacter baumannii* and *Klebsiella pneumoniae* were significantly more frequent in intensive care units, while infections due to various pathogens predominated in pediatric wards.

During the pandemic period, *Clostridioides difficile* infections predominated in medical wards, SARS-CoV-2 infections were more frequent in medical, surgical, and intensive care units, and infections caused by *Acinetobacter baumannii* and other pathogens were more common in intensive care and pediatric wards.

During the post-pandemic period, *Clostridioides difficile* infections predominated in intensive care units, SARS-CoV-2 infections were more frequent in medical wards, and infections caused by *Acinetobacter baumannii* and *Klebsiella pneumoniae* were more commonly identified in intensive care units. Infections due to various pathogens continued to be most frequently observed among patients admitted to pediatric wards.

### 2.8. Distribution of Patients by Discharge Status and Etiology

At discharge, most patients showed clinical improvement (59.7%), while 17.5% died. Clinical improvement was significantly more frequent in the post-pandemic period (67.7% vs. 51.8%), whereas transfers (10.2% vs. 2.3%) and deaths (20.3% vs. 14.6%) were more common during the pandemic period (*p* < 0.001). Among deceased patients, most deaths were not attributed to healthcare-associated infections (59.8%),with this proportion significantly higher in the post-pandemic period (69.5% vs. 53.0%). Deaths with an unknown cause were significantly more frequent during the pandemic period (24.5% vs. 9.3%; *p* < 0.001) (Table 5).

The analysis of the relationship between the etiology of healthcare-associated infections and discharge status showed that patients with *Clostridioides difficile* infection were most frequently associated with both favorable outcomes at discharge and death, whereas patients with SARS-CoV-2 infection were predominantly transferred to other healthcare facilities. Infections caused by *Acinetobacter baumannii* were more frequently associated with mortality, while infections due to various pathogens were mainly observed among patients with a favorable outcome at discharge. These distributions were maintained in both the pandemic and post-pandemic periods, with period-specific variations observed across the analyzed time frames.

### 2.9. Mortality and Factors Associated with HAI-Attributable Death

Data presented in Table 6 summarize the distribution of the analyzed factors according to the adjudicated cause of death. Variables that showed statistically significant differences were subsequently entered into a multivariable binomial logistic regression models. In the final multivariable model, only admission to a medical ward (*p* = 0.024), infection with SARS-CoV-2 (*p* < 0.001), and receipt of antibiotic therapy during the current hospitalization (*p* = 0.005) remained independently associated with the cause of death. Specifically, admission to a medical ward (versus other wards) increased the odds of death being attributed to HAI by 1.93 times (95% CI 1.09–3.43), and infection with SARS-CoV-2 increased these odds by 4.52 times (95% CI 2.16–9.45). In contrast, receipt of antibiotic therapy during admission was associated with a 2.70-fold increase in the odds of death being classified as unrelated to HAI (95% CI 1.35–5.55).

## 3. Discussion

### 3.1. Demographic and Clinical Characteristics

In our study, most patients with healthcare-associated infections (HAIs) were older adults, consistent with previous reports identifying advanced age as a significant risk factor for nosocomial infections due to immunosuppression, multimorbidity, and prolonged hospitalization [9,10]. In contrast, pediatric data suggest that HAI prevalence varies less by age group, with other clinical factors such as comorbidities and exposure to invasive devices playing a more prominent role [11].

The sex distribution in our cohort reflect global epidemiological patterns, with men generally exhibiting slightly higher HAI rates, particularly for respiratory and device-associated infections, compared with women [10,12]. Regional data from oral and maxillofacial surgery further support a higher HAI incidence among male patients, with sex specific differences in infection profiles [13].

Length of stay was clearly associated with HAI occurrence in our cohort, with infected patients experiencing longer hospitalizations than those without HAIs, in line with evidence showing both the role of prolonged stay as a risk factor and the impact of HAIs in further extending hospitalization [14,15]. These observations emphasize the need for early, targeted prevention strategies in high-risk patients who require extended inpatient care [8].

### 3.2. Distribution of Cases Across Hospital Wards

In both analyzed periods, most HAIs were reported in medical wards, where patients often have multiple comorbidities, repeated antibiotic exposure, and a high prevalence of *Clostridioides difficile* infection [6,16,17]. National data from Romania indicate that, in internal medicine departments, a large proportion of HAIs may be due to *C. difficile*, consistent with our observations [16].

During the COVID-19 pandemic, the burden of HAIs shifted towards intensive care units (ICUs), with increased rates of bloodstream infections and ventilator-associated pneumonias in critically ill COVID-19 patients, as documented in several cohorts [18]. In the post-pandemic period, as routine activity progressively resumed, HAIs increasingly reflected the case mix in medical and surgical wards rather than exclusively COVID-19 ICUs, in line with reports on changing organizational patterns and infection profiles [8].

Pediatric wards recorded the lowest HAI burden, predominantly mild respiratory or gastrointestinal infections with short lengths of stay, similar to studies reporting that pediatric HAIs account for a smaller proportion of total nosocomial infections and have lower prevalence than overall hospital rates [19,20].

### 3.3. Types of HAIs During and After the COVID-19 Pandemic

In our cohort, the most frequent HAIs were *Clostridioides difficile* infections, pneumonias, urinary tract infections, bloodstream infections, and surgical site infections, consistent with international epidemiological patterns that highlight respiratory, urinary, bloodstream, surgical site, and gastrointestinal (*C. difficile*) infections as the main HAIs [6,10]. During the pandemic period, bloodstream infections and pneumonias—particularly ventilator-associated pneumonia (VAP) in COVID-19 patients—were more common, reflecting extensive device use and prolonged ICU stays [18,21,22].

Numerous investigations have reported markedly elevated VAP rates in mechanically ventilated COVID-19 patients, along with a prevalence of multidrug-resistant Gram-negative pathogens in ICU-acquired pneumonias and bloodstream infections [18,23]. At the same time, many centers reported a temporary reduction in postoperative wound infections during the pandemic, attributed to fewer elective surgical procedures and enhanced aseptic protocols, a phenomenon also observed in our data [24].

Catheter-associated urinary tract infections and skin and soft-tissue infections were less common in our group, but remained clinically significant as they caused illness and prolonged hospital stays [25]. The overall pattern supports prioritizing the prevention of device-associated infections (vascular and urinary catheters, mechanical ventilation), for which structured, evidence-based prevention bundles are already well established [25].

The very low proportions of bloodstream infections and urinary tract infections among reported HAIs suggest the possibility of under-reporting. As surveillance is based on routine reporting by hospital staff, it is likely that some cases, particularly BSIs and UTIs, remained undetected or unreported, which should be taken into account when interpreting our results.

### 3.4. Etiology of Healthcare-Associated Infections

The etiological profile of HAIs in our study was dominated by *Clostridioides difficile* and multidrug-resistant Gram-negative bacteria, particularly *Acinetobacter baumannii* and *Klebsiella pneumoniae*, as well as *Escherichia coli*, *Pseudomonas aeruginosa*, and Gram-positive cocci such as *Staphylococcus aureus*. This pattern aligns with Romanian and international data, which highlight the central role of these pathogens in HAIs, especially among severely ill patients in high dependency settings [6,26].

*Clostridioides difficile* was the most frequent etiologic agent in both the pandemic and post-pandemic periods, with a marked increase in post-pandemic incidence associated with intense, often prolonged antibiotic use [16,17]. Romanian studies show that *Clostridioides difficile* is the leading etiologic agent of healthcare-associated infections, with a particularly high burden in medical wards, confirming its dominant role in national nosocomial epidemiology [16,17]. The significant correlation between CDI, extensive antibiotic exposure, and recurrent hospitalizations reinforces the need to establish comprehensive antimicrobial stewardship programs and proactive CDI monitoring [17,27].

*Acinetobacter baumannii* and *Klebsiella pneumoniae* were major contributors to bloodstream infections and ventilator-associated pneumonia (VAP) during the pandemic, especially in ICUs treating COVID-19 patients [18,21]. These organisms, known for their high resistance to carbapenems and many other classes of antibiotics, are believed to be responsible for many HAIs linked to devices in the ICU. They also lead to significant mortality and prolonged hospital stays [28]. Their continued presence after the pandemic indicates ongoing transmission concerns and reinforces the need to improve environmental cleanliness, patient isolation, and transmission-based precaution, along with the strict rational use of broad-spectrum antibiotics [6,8].

*Escherichia coli* predominantly causes urinary tract infections and specific intra-abdominal infections, maintaining a relatively stable share of the etiological spectrum despite the extensive use of third-generation cephalosporins and fluoroquinolones, which may contribute to increasing resistance among Enterobacterales [25]. *Pseudomonas aeruginosa* was mainly implicated in respiratory and device-associated infections among ICU patients, consistent with studies highlighting its involvement in ventilator- and catheter-associated infections in critically ill populations, which are frequently characterized by considerable antibiotic resistance [23].

*Staphylococcus aureus*, including methicillin-resistant strains (MRSA), was a less prevalent cause of healthcare-associated infections (HAIs) in our sample, primarily associated with surgical site and skin and soft tissue infections, yet with significant clinical implications. During the pandemic, reductions in elective surgical procedures and the implementation of enhanced hygiene precautions were associated with a temporary decline in surgical site infection rates in many centers, followed by a gradual increase as surgical activity resumed, a pattern also observed at our institution [24].

The “other pathogens” category included coagulase-negative staphylococci, *Enterococcus* spp., and *Candida* spp., which were mainly responsible for bloodstream infections and infections in patients with severe comorbidities or invasive devices [29]. Although each organism constitutes a small proportion of healthcare-associated infections (HAIs), together they increase the complexity of the etiological landscape and demonstrate the impact of invasive procedures and antibiotic exposure on hospital microbiota [29].

### 3.5. Clinical Progress and Discharge Status

In our cohort, the majority of patients with HAIs were discharged with positive outcomes (clinical cure or improvement); nevertheless, a significant number experienced stagnant progression, clinical deterioration, or mortality during hospitalization [29]. Multicentre studies have shown that HAIs increase length of stay and mortality, especially for bloodstream infections and device-associated pneumonias in critically ill patients [8].

Our results indicate that infections caused by multidrug-resistant organisms, particularly *A. baumannii* and *K. pneumoniae*, were more frequently associated with adverse outcomes and in-hospital mortality, consistent with evidence linking multidrug-resistant healthcare-associated infections to increased mortality and prolonged hospitalization [21,29]. These findings underscore the importance of antimicrobial stewardship programs and rigorous infection control protocols to prevent the spread of multidrug-resistant bacteria within hospital environments.

### 3.6. Relationship Between HAIs and In-Hospital Mortality

In the analyzed cohort, a significant proportion of patients with HAIs died during hospitalization, and HAIs were associated with higher in-hospital mortality compared with patients without [29]. This distribution highlights the challenge of distinguishing the contribution of HAIs from that of severe underlying comorbidities and baseline illness, a difficulty also reported in population-based studies assessing infection-related mortality and cause-of-death coding for procedure- and device-related HAIs [30].

Standardized tools for classifying the relationship between HAIs and death have been developed to support mortality surveillance, but attributing deaths to infection versus underlying disease remains challenging. Incorporating such standardized assessment tools into routine practice could improve understanding of the actual impact of HAIs on in-hospital mortality and help prioritize prevention interventions.

### 3.7. Risk Factors for HAIs and Clostridioides Difficile Infection

In our study, key risk factors for HAIs, particularly for CDI, included immunosuppression, recent surgical interventions, prior hospitalizations, and antibiotic exposure during the current admission [16]. This risk profile is consistent with international evidence identifying the same variables as significant determinants of HAI risk and CDI recurrence [31].

Immunosuppression was more frequent in the post-pandemic period, reflecting increased use of corticosteroids, biologic therapies, and chemotherapy. Immunocompromised patients had a significantly higher risk of HAIs and a prolonged clinical course. Recent hospitalizations, particularly within the preceding months, were an essential predictor of CDI onset and recurrence, due to cumulative exposure to the hospital environment and repeated antibiotic courses.

The main modifiable risk factor for CDI-related HAIs was antibiotic exposure during hospitalization, with risk increasing according to both the quantity and variety of antibiotic classes administered. Our findings align with research indicating increased antibiotic use among hospitalized patients and its role in worsening healthcare-associated infections (HAIs) and antimicrobial resistance, highlighting the need for stewardship interventions [1].

### 3.8. Exposure to Antibiotics, Concurrent Treatments, and the Diagnosis of CDI

Our data confirm that both antibiotic treatment during the present hospitalization and recent outpatient antibiotic exposure have a cumulative effect on CDI risk, maintaining patient susceptibility for several weeks after therapy. Previous studies have shown that exposure to fluoroquinolones, third-generation cephalosporins, and beta-lactam/beta-lactamase inhibitor combinations is associated with a significantly increased risk of CDI, particularly in older patients and those with repeated hospital admissions [31].

Concomitant therapies with immunosuppressive agents, proton pump inhibitors, and chemotherapy further increase CDI risk by altering the intestinal microbiota and host defence mechanisms [32]. In addition, contact with known CDI cases and environmental contamination in multi-bed wards represent relevant pathways of spread, although documented contact history was recorded in only a small proportion of our patients.

Current CDI diagnosis in clinical practice relies on the detection of toxins and/or toxin genes using immunoassays and molecular methods, in accordance with guideline-recommended two-step algorithms [33]. The use of standardized diagnostic criteria is essential to distinguish colonization from active disease, maintain accurate HAIs reporting, and enable timely implementation of transmission-based infection control measures.

### 3.9. Limitations

One limitation of our study is the pragmatic definition of the “post-pandemic” period, which is based on the timing of the lifting of legal restrictions rather than on strict epidemiological criteria. This approach primarily reflects administrative and public health policy changes and may not fully capture the actual transition in SARS-CoV-2 transmission dynamics and population behaviour.

A key limitation of this study is that it relies on reported surveillance data rather than on active, uniform screening of all hospital admissions, which may lead to under-ascertainment or variability in case detection between hospitals. In addition, passive surveillance is inherently vulnerable to differences in awareness, workload, diagnostic resources and local reporting practices, which may introduce selection and information bias and limit the comparability of HAIs rates across institutions.

Detailed information on specific resistance mechanisms (e.g., ESBL, carbapenemases) and full susceptibility panels was not available in the source dataset, which precluded formal analysis of resistance profiles and trends. This constraint limits our ability to draw detailed conclusions about antimicrobial resistance dynamics and underscores the need for future studies with standardized, microbiology-rich surveillance systems.

## 4. Materials and Methods

### 4.1. Study Design and Population

This study was designed as a retrospective observational analysis based on surveillance data reported to the County Public Health Directorate from all healthcare facilities in the county, rather than on active, uniform screening of all hospital admissions. Data were collected from eight public hospitals and two private hospitals. Only patients with confirmed healthcare-associated infections (HAIs) were included in the analysis; these patients were registered in the national surveillance system and reported by local hospitals during the study period.

The study period covered March 2020 to December 2024, allowing comparison between the pandemic period (March 2020–March 2022) and the post-COVID period (April 2022–December 2024), the latter beginning with the lifting of the national state of alert, after which restriction measures were no longer applied, in accordance with Romanian legislation. A total of 3929 hospitalized patients were included, selected based on the eligibility criterion of clinical or microbiological documentation of an HAI.

Cases identified in the Emergency Department (ED) were included only if patients had been hospitalized within the previous 28 days; in these instances, infections were classified as HAIs according to national surveillance criteria and included in the final analysis.

Exclusion criteria targeted patients reported from residential care facilities for older adults, as well as those with incomplete standardized reporting forms or with previous infectious episodes not attributable to the current hospitalization. This methodological approach enabled the creation of a representative county-level cohort, providing a solid basis for analyzing clinical, epidemiological, and microbiological factors.

Healthcare-associated infections were defined according to the National Methodology for HAIs Surveillance [34] and were consistent with case definitions established by the European Centre for Disease Prevention and Control (ECDC) [34]. For terminological consistency, the term HAIs is used throughout the manuscript as a general designation.

### 4.2. Variables Analyzed

For each patient, sociodemographic, clinical, therapeutic, and microbiological data were collected. Demographic variables included age, sex, and area of residence. The clinical profile comprised the type of hospital ward (ICU, medical, surgical, pediatric, ED), length of hospitalization, relevant comorbidities, and immunosuppressive status.

Prior exposures were recorded, including hospitalizations within the past year, antibiotic treatments during the current admission and within the previous three months, administration of immunosuppressive drugs, gastric antisecretory therapy, chemotherapy, and previous surgical interventions.

For etiology, HAIs diagnoses were recorded (including *Clostridioides difficile* infections, respiratory infections, urinary tract infections, surgical site infections, skin infections, etc.), isolated pathogens (SARS-CoV-2, *Clostridioides difficile*, *Acinetobacter baumannii*, *Klebsiella pneumoniae*, *Escherichia coli*, *Staphylococcus aureus*, *Pseudomonas aeruginosa*, and other pathogens), and, where available, results of specific tests for *Clostridioides difficile* (toxins A/B, PCR, presence of pseudomembranous colitis, histopathological examination).

In the present study, detailed microbiological data on specific resistance mechanisms in Gram-negative bacteria (e.g., ESBL, KPC and other carbapenemases), as well as information on prior antibiotic exposure (class, specific agent and duration) before *Clostridioides difficile* infection onset, were not available in the source dataset. Consequently, we were not able to evaluate temporal trends in carbapenem resistance profiles or to assess associations between particular antibiotics and subsequent CDI.

The primary outcome variable was clinical status at discharge (recovered, improved, stable, transferred, deceased). For deceased patients, the cause of death was classified as HAI-related, non-HAI-related, or unknown.

### 4.3. Statistical Analysis

Data processing and analysis were performed using IBM SPSS Statistics 25, while graphs and tables were generated with Microsoft Office Excel and Word 2024.

Qualitative variables were presented as counts or percentages and compared between groups using Fisher’s exact test. To detail the differences observed in the contingency tables, Z-tests with Bonferroni correction were applied.

Quantitative variables were expressed as means ± standard deviations or as medians with interquartile ranges. The normality of distribution for quantitative variables was assessed using the Shapiro–Wilk test. Independent quantitative variables with non-parametric distribution were compared between groups using the Mann–Whitney U test. The level of significance was set at α = 0.05 for all tests.

### 4.4. Aims of the Study

This study aimed to characterize and compare healthcare-associated infections during and after the COVID-19 pandemic in southeastern Romania, to identify etiological patterns, risk factors, and their impact on clinical outcomes.

### 4.5. Statement of Ethics

This study did not require ethical approval as it was a retrospective observational analysis of anonymized secondary data that did not include any personal information. The investigation was conducted in compliance with national legislation governing the use of anonymised data for HAI surveillance.

## 5. Conclusions

The study identified a substantial and ongoing burden of healthcare-associated infections (HAIs) in southeastern Romania. These primarily affected older patients with prolonged hospital stays and complex clinical conditions, leading to notable overall mortality. Most cases occurred in medical wards. Intensive care units experienced an increased burden during the pandemic period. In contrast, pediatric wards reported few HAIs, mainly mild respiratory or digestive infections.

*Clostridioides difficile* emerged as the most common etiological agent, with increased prevalence in the period following the pandemic. Its occurrence was associated with repeated hospitalizations, cumulative antibiotic exposure at admission and in previous months, antisecretory therapy, chemotherapy, and immunosuppression. Infections due to *Acinetobacter baumannii* and *Klebsiella pneumoniae* were concentrated in intensive care units and more often resulted in adverse outcomes and mortality. The results suggest ongoing transmission of multidrug-resistant Gram-negative bacilli in settings with critically ill patients and frequent use of invasive devices.

Comparison of the pandemic and post-pandemic periods revealed a shift from a profile characterized by severe COVID-19 cases, intensive care unit admissions, pneumonias, and bloodstream infections, which were associated with higher mortality and often ambiguous causes of death. In the post-pandemic period, *Clostridioides difficile* infection and immunocompromised patients became more prominent. Although mortality decreased, it remained considerable and was frequently associated with comorbidities and complex therapeutic regimens.

These observations highlight the need to strengthen antimicrobial stewardship programs by minimizing unwarranted and prolonged use of broad-spectrum antibiotics, enhancing infection-prevention strategies for device-associated infections in intensive care units, and consistently applying prevention bundles. Optimizing *Clostridioides difficile* infection diagnosis and surveillance, improving the accuracy of HAI reporting, and clarifying death attribution may provide more precise estimates of the true impact of HAIs and inform the development of targeted measures to reduce morbidity and mortality in Romanian hospitals.

## Figures and Tables

**Table 1:** Comparison of demographic characteristics, causes of death, and risk factors according to the analyzed admission periods.

Parameter (N, %)	Total	Pandemic	Post-Pandemic	*p*
N, %	3929 (100%)	1754 (44.6%)	2175 (55.4%)	**-**
*Age (Median (IQR))*	67 (55–76)	66 (55–75)	68 (55–76)	0.260 **
*Gender (Male)*	2051 (52.2%)	961 (54.8%)	1090 (50.1%)	0.004 *
*Environment (Urban)*	2722 (69.4%)	1246 (71.2%)	1476 (69.4%)	0.028 *
*Hospitalization period (Median (IQR))*	15 (10–22)	16 (10–24)	14 (9–21)	<0.001 *
*Discharge status*				
Cured	375 (12%)	200 (12.7%)	175 (11.3%)	<0.001 *
Improved	1864 (59.7%)	814 (51.8%)	1050 (67.7%)	<0.001 *
Stationary	125 (4%)	69 (4.4%)	55 (3.5%)	<0.001 *
Worsened	16 (0.5%)	8 (0.5%)	8 (0.5%)	<0.001 *
Transferred	196 (6.3%)	160 (10.2%)	36 (2.3%)	<0.001 *
Deceased	545 (17.5%)	319 (20.3%)	226 (14.6%)	<0.001 *
*Cause of death*				
Not related to HAIs	326 (59.8%)	169 (53%)	157 (69.5%)	<0.001 *
Possibly related to HAIs	82 (15%)	49 (15.4%)	33 (14.6%)	<0.001 *
HAIs	38 (7%)	23 (7.2%)	15 (6.6%)	<0.001 *
Unknown	99 (18.2%)	78 (24.5%)	21 (9.3%)	<0.001 *
*HAI risk factors*				
Immunosuppression	534 (13.6%)	60 (3.4%)	474 (21.8%)	<0.001 *
Surgical intervention	346 (8.8%)	69 (3.9%)	277 (12.7%)	<0.001 *

* Fisher’s Exact Test, ** Mann–Whitney U Test.

**Table 2:** Comparison of section type, HAI type, and etiology according to the analyzed admission periods.

Parameter (N, %)	Total	Pandemic	Post-Pandemic	*p*
N, %	3929 (100%)	1754 (44.6%)	2175 (55.4%)	-
*Section type*				
ICU	163 (4.1%)	106 (6%)	57 (2.6%)	<0.001 *
Medical	3061 (77.9%)	1339 (76.3%)	1722 (79.2%)	<0.001 *
Surgical	574 (14.6%)	251 (14.3%)	323 (14.9%)	<0.001 *
Pediatric	109 (2.8%)	51 (2.9%)	58 (2.7%)	<0.001 *
Emergency department	22 (0.6%)	7 (0.4%)	15 (0.7%)	<0.001 *
*HAI type*				
Septicemia	23 (0.6%)	13 (0.7%)	10 (0.5%)	<0.001 *
Surgical site infections	73 (1.9%)	0 (0%)	73 (3.4%)	<0.001 *
CVC-associated infections	3 (0.1%)	3 (0.2%)	0 (0%)	<0.001 *
PIVC-associated infections	2 (0.1%)	1 (0.1%)	1 (0.1%)	<0.001 *
Pneumonia	28 (0.7%)	28 (1.6%)	0 (0%)	<0.001 *
SARS-CoV-2	1306 (33.2%)	652 (37.2%)	654 (30.1%)	<0.001 *
UTI	108 (2.7%)	52 (3%)	56 (2.6%)	<0.001 *
Upper respiratory tract infections	36 (0.9%)	33 (1.9%)	3 (0.1%)	<0.001 *
Lower respiratory other than pneumonia	25 (0.6%)	4 (0.2%)	21 (1%)	<0.001 *
Other lower respiratory tract infections	70 (1.8%)	31 (1.8%)	39 (1.8%)	<0.001 *
*Clostridioides difficile* infections	2128 (54.2%)	884 (50.4%)	1244 (57.2%)	<0.001 *
Gastroenteritis (except CDI)	73 (1.9%)	16 (0.9%)	57 (2.6%)	<0.001 *
Gastrointestinal infections (except CDI, GE)	7 (0.2%)	7 (0.4%)	0 (0%)	<0.001 *
Intra-abdominal infections	4 (0.1%)	3 (0.2%)	1 (0.1%)	<0.001 *
Arterial or venous infections	2 (0.1%)	2 (0.1%)	0 (0%)	<0.001 *
Systemic infections	1 (0.1%)	0 (0%)	1 (0.1%)	<0.001 *
Disseminated infections	3 (0.1%)	3 (0.2%)	0 (0%)	<0.001 *
Infections of the female/male reproductive tract	4 (0%)	1 (0.1%)	3 (0.1%)	<0.001 *
Central nervous system infections	4 (0.1%)	2 (0.1%)	2 (0.1%)	<0.001 *
Skin infections	2 (0.1%)	0 (0%)	2 (0.1%)	<0.001 *
Skin and soft tissue infections	27 (0.7%)	19 (1.1%)	8 (0.4%)	<0.001 *
*Etiology*				
Unique	3817 (97.1%)	1708 (97.4%)	2109(97%)	0.500 * 0.500 *
Multiple	112 (2.9%)	46 (2.6%)	66 (3%)	
*Etiology—Type of germ*				
*Clostridioides difficile*	2164 (55.1%)	896 (51.1%)	1268 (58.3%)	<0.001 *
SARS-CoV-2	1306 (33.2%)	652 (37.2%)	654 (30.1%)	<0.001 *
*Acinetobacter baumannii*	77 (2%)	39 (2.2%)	38 (1.7%)	0.299 *
*Klebsiella pneumoniae*	76 (1.9%)	36 (2.1%)	40 (1.8%)	0.643 *
*Escherichia coli*	53 (1.3%)	21 (1.2%)	32 (1.5%)	0.490 *
*Staphylococcus aureus*	39 (1%)	17 (1%)	22 (1%)	1.000 *
*Pseudomonas aeruginosa*	35 (0.9%)	17 (1%)	18 (0.8%)	0.733 *
Other germs	296 (7.5%)	121 (6.9%)	175 (8%)	0.182 *

* Fisher’s Exact Test.

**Table 3:** Comparison of risk factors for CDI patient risk across the analyzed admission periods.

Parameter (N, %)	Total	Pandemic	Post-Pandemic	*p*
N, %	3929 (100%)	1754 (44.6%)	2175 (55.4%)	**-**
*CDI—Hospitalization within last year* (N = 1052)				
<4 weeks ago	793 (75.4%)	277 (74.3%)	516 (76%)	0.759 *
4–12 weeks ago	160 (15.2%)	58 (15.5%)	102 (15%)	0.759 *
>12 weeks ago	99 (9.4%)	38 (10.2%)	61 (9%)	0.759 *
Antibiotic treatment—Current admission	1269 (32.3%)	631 (36%)	638 (29.3%)	<0.001 *
*Antibiotic treatment—Current admission*				
One session, one antibiotic	447 (35.2%)	199 (31.5%)	248 (38.9%)	0.006 *
Multiple sessions, one antibiotic	316 (24.9%)	177 (28.1%)	139 (21.8%)	0.006 *
One session, multiple antibiotics	230 (18.1%)	107 (17%)	123 (19.3%)	0.006 *
Multiple sessions, multiple antibiotics	276 (21.7%)	148 (23.5%)	128 (20.1%)	0.006 *
Antibiotic treatment—Last 3 months	631 (16.1%)	300 (17.1%)	331 (15.2%)	0.116 *
*Antibiotic treatment—Last 3 months*				
One session, one antibiotic	250 (39.6%)	108 (36%)	142 (42.9%)	0.271 *
Multiple sessions, one antibiotic	128 (20.3%)	63 (21%)	65 (19.6%)	0.271 *
One session, multiple antibiotics	126 (20%)	61 (20.3%)	65 (19.6%)	0.271 *
Multiple sessions, multiple antibiotics	127 (20.1%)	68 (22.7%)	59 (17.8%)	0.271 *
*Treatment in the last 3 months*				
Immunosuppressant drugs	119 (3%)	70 (4%)	49(2.3%)	0.002 *
Antisecretory drugs	1083 (27.6%)	387 (22.1%)	696 (32%)	<0.001 *
Chemotherapy	60(1.5%)	13 (0.7%)	47 (2.2%)	<0.001 *
*Contact—CDI case* (N = 2128)	38 (1.8%)	32 (3.6%)	6 (0.5%)	<0.001 *
*Laboratory tests for CDI*				
A/B toxin positive (N = 2128)	2079 (97.7%)	875 (99%)	1204 (96.8%)	0.001 *
PCR positive (N = 2128)	141 (6.6%)	30 (3.4%)	111 (8.9%)	<0.001 *
Colonoscopy—Pseudomembranous colitis appearance (N = 2128)	9 (0.2%)	2 (0.1%)	7 (0.3%)	0.314 *
Histopathological exam (N = 2128)	2 (0.1%)	0 (0%)	2 (0.1%)	0.506 *

* Fisher’s Exact Test, Mann-Whitney U Test.

**Table 4:** Distribution of patients (total study group, pandemic admission, post-pandemic admissions) according to section type and etiology.

Total Study Group						
Section/Etiology	ICU (Nr.,%)	Medical (Nr.,%)	Surgical (Nr.,%)	Pediatric (Nr.,%)	Emergency (Nr.,%)	*p* *
*Clostridioides difficile*	79	1718	324	24	19	
	(48.5%)	(56.1%)	(56.4%)	(22%)	(86.4%)	<0.001
SARS-CoV-2	8	1001	160	7	3	<0.001
	(4.9%)	(32.7%)	(27.9%)	(6.4%)	(13.6%)	
*Acinetobacter baumannii*	28	33	10	6	0	<0.001
	(17.2%)	(1.1%)	(1.7%)	(5.5%)	(0%)	
*Klebsiella pneumoniae*	18	38	14	6	0	<0.001
	(11%)	(1.2%)	(2.4%)	(5.5%)	(0%)	
*Escherichia coli*	1 (0.6%)	31 (1%)	19 (3.3%)	2 (1.8%)	0 (0%)	0.002
*Staphylococcus aureus*	5 (3.1%)	24 (0.8%)	7 (1.2%)	3 (2.8%)	0 (0%)	0.021
*Pseudomonas aeruginosa*	5 (3.1%)	24 (0.8%)	5 (0.9%)	1 (0.9%)	0 (0%)	0.096
Other germs	41 (25.2%)	271 (8.9%)	53 (9.2%)	63 (57.8%)	0 (0%)	<0.001
**Pandemic admission**						
*Clostridioides difficile*	48	713	114	17	4	
	(45.3%)	(53.2%)	(45.4%)	(33.3%)	(57.1%)	<0.007
SARS-CoV-2	5	436	888	3	3	<0.001
	(4.7%)	(32.6%)	(35.1%)	(5.9%)	(42.9%)	
*Acinetobacter baumannii*	16	14	4	5	0	<0.001
	(15.1%)	(1%)	(1.6%)	(9.8%)	(0%)	
*Klebsiella pneumoniae*	11	15	6	4	0	<0.001
	(10.4%)	(1.1%)	(2.4%)	(7.8%)	(0%)	
*Escherichia coli*	1 (0.9%)	10 (0.7%)	8 (3.2%)	2 (3.9%)	0 (0%)	0.009
*Staphylococcus aureus*	4 (3.8%)	10 (0.7%)	2 (0.8%)	1 (2%)	0 (0%)	0.052
*Pseudomonas aeruginosa*	3 (2.8%)	13 (1%)	1 (0.4%)	0 (0%)	0 (0%)	0.278
Other germs	31 (29.2%)	155 (11.6%)	33 (13.1%)	21 (14.2%)	0 (0%)	<0.001
**Post-pandemic admission**						
*Clostridioides difficile*	31	1005	210	7	15	
	(54.4%)	(58.4%)	(65%)	(12.1%)	(100%)	<0.001
SARS-CoV-2	3	565	72	4	0	<0.001
	(5.3%)	(32.8%)	(22.3%)	(6.9%)	(0%)	
*Acinetobacter baumannii*	12	19	6	1	0	<0.001
	(21.1%)	(1.1%)	(1.9%)	(1.7%)	(0%)	
*Klebsiella pneumoniae*	7	23	8	2	0	<0.001
	(12.3%)	(1.3%)	(2.5%)	(3.4%)	(0%)	
*Escherichia coli*	0 (0%)	21 (1.2%)	11 (3.4%)	0 (0%)	0 (0%)	0.085
*Staphylococcus aureus*	1 (1.8%)	14 (0.8%)	5 (1.5%)	2 (3.4%)	0 (0%)	0.128
*Pseudomonas aeruginosa*	2 (3.5%)	11 (0.6%)	4 (1.2%)	1 (1.7%)	0 (0%)	0.081
Other germs	10 (17.5%)	116 (6.7%)	20 (6.2%)	42 (72.4%)	0 (0%)	<0.001

* Fisher’s Exact Test.

**Table 5:** Distribution of patients (post-pandemic admissions) according to discharge status and etiology.

Total Study Group							
Status/Etiology	Cured (Nr.,%)	Improved (Nr.,%)	Stationary (Nr.,%)	Worsened (Nr.,%)	Transfer (Nr.,%)	Deceased (Nr.,%)	*p* *
*Clostridioides difficile*	226	994	52	10	29	323	<0.001
	(60.3%)	(53.3%)	(41.9%)	(62.5%)	(14.8%)	(59.2%)	
SARS-CoV-2	61	622	58	3	138	130	<0.001
	(16.3%)	(33.4%)	(46.8%)	(18.8%)	(70.4%)	(23.8%)	
*Acinetobacter baumannii*	6	17	3	1	2	33	<0.001
	(1.6%)	(0.9%)	(2.4%)	(6.3%)	(1%)	(6%)	
*Klebsiella pneumoniae*	5	33	1	1	1	16	0.125
	(1.3%)	(1.8%)	(0.8%)	(6.3%)	(0.5%)	(2.9%)	
*Escherichia coli*	11 (2.9%)	26 (1.4%)	0 (0%)	0 (0%)	1 (0.5%)	4 (0.7%)	0.082
*Staphylococcus aureus*	6 (1.6%)	19 (1%)	2 (1.6%)	0 (0%)	3 (1.5%)	6 (1.1%)	0.719
*Pseudomonas aeruginosa*	6 (1.6%)	18 (1%)	0 (0%)	0 (0%)	3 (1.5%)	2 (0.4%)	0.298
Other germs	63 (16.8%)	185 (9.9%)	14 (11.3%)	2 (12.5%)	22 (11.2%)	62 (11.4%)	0.013
**Pandemic admission**							
*Clostridioides difficile*	128	476	19	4	9	167	
	(64%)	(58.5%)	(27.5%)	(50%)	(5.6%)	(52.4%)	<0.001
SARS-CoV-2	22	208	39	2	124	75	<0.001
	(11%)	(25.6%)	(56.5%)	(25%)	(77.5%)	(23.5%)	
*Acinetobacter baumannii*	5	8	2	0	2	20	<0.001
	(2.5%)	(1%)	(2.9%)	(0%)	(1.3%)	(6.3%)	
*Klebsiella pneumoniae*	3	16	0	1	1	12	0.060
	(1.5%)	(2%)	(0%)	(12.5%)	(0.6%)	(3.8%)	
*Escherichia coli*	7 (3.5%)	9 (1.1%)	0 (0%)	0 (0%)	1 (0.6%)	4 (1.3%)	0.188
*Staphylococcus aureus*	3 (1.5%)	9 (1.1%)	0 (0%)	0 (0%)	2 (1.3%)	3 (0.9%)	0.941
*Pseudomonas aeruginosa*	6 (3%)	6 (0.7%)	0 (0%)	0 (0%)	3 (1.9%)	1 (0.3%)	0.051
Other germs	32 (16%)	99 (12.2%)	11 (15.9%)	2 (25%)	21 (13.1%)	50 (15.7%)	0.345
**Post-pandemic admission**							
*Clostridioides difficile*	98	518	33	6	20	156	
	(56%)	(49.3%)	(60%)	(75%)	(55.6%)	(68.7%)	<0.001
SARS-CoV-2	39	414	19	1	14	55	<0.001
	(22.3%)	(39.4%)	(34.5%)	(12.5%)	(38.9%)	(24.2%)	
*Acinetobacter baumannii*	1	9	1	1	0	13	<0.001
	(0.6%)	(0.9%)	(1.8%)	(12.5%)	(0%)	(5.7%)	
*Klebsiella pneumoniae*	2	17	1	0	0	4	0.938
	(1.1%)	(1.6%)	(1.8%)	(0%)	(0%)	(1.8%)	
*Escherichia coli*	4 (2.3%)	17 (1.6%)	0 (0%)	0 (0%)	0 (0%)	0 (0%)	0.275
*Staphylococcus aureus*	3 (1.7%)	10 (1%)	2 (3.6%)	0 (0%)	1 (2.8%)	3 (1.3%)	0.219
*Pseudomonas aeruginosa*	0 (0%)	12 (1.1%)	0 (0%)	0 (0%)	0 (0%)	1 (0.4%)	0.677
Other germs	31 (17.7%)	86 (8.2%)	3 (5.5%)	0 (0%)	1 (2.8%)	12 (5.3%)	0.001

* Fisher’s Exact Test.

**Table 6:** Multivariable binomial logistic regression models used for the prediction of causes of death.

Parameter	Multivariable	
	OR (95% C.I.)	*p*
Medical section	1.93 (1.09–3.43)	0.024
Clostridium difficile	1.45 (0.61–3.46)	0.392
SARS-CoV-2	4.52 (2.16–9.45)	<0.001
AB-Admission	0.37 (0.18–0.74)	0.005
Last 3M-AB	0.68 (0.30–1.51)	0.346
Last 3M-Antisecretory	0.90 (0.45–1.79)	0.766

## Data Availability

The original contributions presented in this study are included in the article. Further inquiries can be directed to the corresponding author.

## Abbreviations

The following abbreviations are used in this manuscript:

HAIs	Healthcare-associated infections
IQR	Interquartile range
*p*	*p*-value
ICU	Intensive care unit
CVC-associated infections	Central venous catheter-associated infections
PIVC-associated infections	Peripheral intravenous catheter-associated infections
CDI	Clostridioides difficile infection
GE	Gastroenteritis
UTI	Urinal tract infection
ED	Emergency department
PCR	Polymerase chain reaction
ECDC	European Centre for Disease Prevention and Control
IBM	International Business Machines
SPSS	Statistical Package for the Social Sciences
MDR infections	Multidrug-resistant infections
spp	Species
SARS-CoV-2	Severe Acute Respiratory Syndrome Coronavirus 2
COVID-19	Coronavirus Disease 2019
WHO	World Health Organization
AB	Antibiotherapy
Last 3M	Last 3 months

## References

[1] Abubakar U., Awaisu A., Khan A.H., Alam K. (2023). Impact of COVID-19 pandemic on healthcare-associated infections: A systematic review and meta-analysis. Antibiotics.

[2] Dobrović K., Škrobo T., Selec K., Jelić M., Čivljak R., Peršec J., Sakan S., Bušić N., Mihelčić A., Hleb S. (2023). Healthcare-associated bloodstream infections due to multidrug-resistant Acinetobacter baumannii in a COVID-19 intensive care unit: A single-center retrospective study. Microorganisms.

[3] Mateescu D.M., Ilie A.C., Cotet I., Guse C., Muresan C.O., Pah A.M., Badalica-Petrescu M., Iurciuc S., Craciun M.-L., Avram A. (2025). Global burden of bloodstream infections in COVID-19: Prevalence, antimicrobial resistance, and mortality risk. Viruses.

[4] Coman A., Pop D., Mureșan F., Oprescu F., Fjaagesund S. (2025). Surveillance and reporting of hospital-associated infections: A document analysis of Romanian healthcare legislation evolution over 20 years. Healthcare.

[5] Hashad N., Stewart D., Perumal D., Abdulrazzaq N., Tonna A.P. (2022). Impact of COVID-19 on antimicrobial stewardship programme implementation in hospitals: An exploration informed by the Consolidated Framework for Implementation Research. J. Hosp. Infect..

[6] Voinea C., Mocanu E., Dantes E., Jurja S., Neculai A.M., Crăciun A., Rugina S. (2025). Burden of healthcare-associated infections on mortality among COVID-19 hospitalized patients. J. Clin. Med..

[7] Montrucchio G., Grillo F., Balzani E., Gavanna G., Sales G., Bonetto C., Simonetti U., Zanierato M., Fanelli V., Filippini C. (2025). Impact of multidrug-resistant bacteria in a cohort of COVID-19 critically ill patients: Data from a prospective observational study conducted in a high-antimicrobial-resistance-prevalence center. J. Clin. Med..

[8] Voinea C., Mocanu E., Opariuc-Dan C., Dantes E., Gache A.C., Rugina S. (2025). Global lessons from COVID-19: Regional variations in the management of hospital-acquired infections during and post-pandemic. J. Clin. Med..

[9] Isigi S.S., Parsa A.D., Alasqah I., Mahmud I., Kabir R. (2023). Predisposing factors of nosocomial infections in hospitalized patients in the United Kingdom: Systematic review. JMIR Public Health Surveill..

[10] Raoofi S., Pashazadeh Kan F., Rafiei S., Hosseinipalangi Z., Noorani Mejareh Z., Khani S., Abdollahi B., Talab F.S., Sanaei M., Zarabi F. (2023). Global prevalence of nosocomial infection: A systematic review and meta-analysis. PLoS ONE.

[11] Moolasart V., Srijareonvijit C., Charoenpong L., Kongdejsakda W., Anugulruengkitt S., Kulthanmanusorn A., Thienthong V., Usayaporn S., Kaewkhankhaeng W., Rueangna O. (2024). Prevalence and risk factors of healthcare-associated infections among hospitalized pediatric patients: A point prevalence survey in Thailand, 2021. Children.

[12] Dias S.P., Brouwer M.C., van de Beek D. (2022). Sex and gender differences in bacterial infections. Infect. Immun..

[13] Bârlean M.C., Scutariu M.M., Crețu C.I., Bobu L., Platon A.L., Tărăboanță-Gamen A., Macovei G. (2023). Gender profile of healthcare-associated infections in Oral and Maxillofacial Surgery Clinic Iași (2011–2018). Rom. J. Oral Rehabil..

[14] Stewart S., Robertson C., Pan J., Kennedy S., Haahr L., Manoukian S., Mason H., Kavanagh K., Graves N., Dancer S. (2021). Impact of healthcare-associated infection on length of stay. J. Hosp. Infect..

[15] Bedada A.T., Imana R.Y., Wodajeneh H.B. (2025). Prevalence and associated factors of hospital-acquired infection among surgical patients at Adama Hospital Medical College, Ethiopia. Sci. Rep..

[16] Iancu D., Moldovan I., Țilea B., Voidăzan S. (2023). Evaluating healthcare-associated infections in public hospitals: A cross-sectional study. Antibiotics.

[17] Vasile C.C., Gheorghe L.A., Chivu C.D., Anghel M.A.M., Mîinea Ș.E., Pițigoi D., Crăciun M.-D. (2024). Clostridioides difficile infections and antibiotherapy: Results of four years of observation in a Romanian tertiary hospital. Microorganisms.

[18] Despotovic A., Milosevic B., Cirkovic A., Vujovic A., Cucanic K., Cucanic T., Stevanovic G. (2021). Impact of COVID-19 on the profile of hospital-acquired infections in adult intensive care units. Antibiotics.

[19] Martins C., Lima D., Cortez Ferreira M., Verdelho Andrade J., Dias A. (2025). Healthcare-associated infections in pediatric patients: A decade of experience in an intensive care unit. Acta Med. Port..

[20] Dashti A.S., Kadivar M.R., Tabatabai A., Zand F., Salami S., Ezadpanah S., Shirvani F., Seifi K., Zarei N. (2019). Prevalence of healthcare-associated infections in pediatric wards of Namazee Teaching Hospital in Shiraz: A Comparison with the Whole Hospital. Arch. Pediatr. Infect. Dis..

[21] Gajic I., Jovicevic M., Popadic V., Trudic A., Kabic J., Kekic D., Ilic A., Klasnja S., Hadnadjev M., Popadic D. (2023). Emergence of multidrug-resistant bacteria causing healthcare-associated infections in COVID-19 patients: A retrospective multicentre study. J. Hosp. Infect..

[22] Hlinkova S., Moraucikova E., Lesnakova A., Strzelecka A., Littva V. (2023). Central line associated bloodstream infections in critically ill patients during and before the COVID-19 pandemic. Healthcare.

[23] Wicky P.H., Dupuis C., Cerf C., Siami S., Cohen Y., Laurent V., Mourvillier B., Reignier J., Goldgran-Toledano D., Schwebel C. (2023). Ventilator-associated pneumonia in COVID-19 patients admitted to intensive care units: Relapse, therapeutic failure and attributable mortality—A multicentric observational study from the OutcomeRea Network. J. Clin. Med..

[24] Ganam S., Sher T., Assy R., Bickel A., Khoury A., Ronit L., Kakiashvili E. (2024). Assessing the impact of enhanced hygiene precautions during the COVID-19 pandemic on surgical site infection risk in abdominal surgeries. BMC Surg..

[25] Werneburg G.T. (2022). Catheter-associated urinary tract infections: Current challenges and future prospects. Res. Rep. Urol..

[26] Alshammari I.T., Alruwaili Y. (2025). Prevalence, microbiological profile, and risk factors of healthcare-associated infections in intensive care units: A retrospective study in Aljouf, Saudi Arabia. Microorganisms.

[27] Ponce-Alonso M., Sáez de la Fuente J., Rincón-Carlavilla A., Moreno-Núñez P., Martínez-García L., Escudero-Sánchez R., Pintor S., García-Fernández S., Cobo J. (2021). Impact of the coronavirus disease 2019 (COVID-19) pandemic on nosocomial Clostridioides difficile infection. Infect. Control. Hosp. Epidemiol..

[28] Kafazi A., Apostolopoulou E., Andreou E., Gavala A., Stefanidis E., Antoniadou F., Stylianou C., Katsoulas T., Myrianthefs P. (2025). Device-associated infections in adult intensive care units: A prospective surveillance study. Acta Microbiol. Hell..

[29] Hema A., Somé S.A., Kaboré O., Sanou S., Poda A., Meda Z.C., Ouedraogo A.S., Savadogo L. (2025). Risk and outcomes of healthcare-associated infections in three hospitals in Bobo Dioulasso, Burkina Faso, 2022: A longitudinal study. PLoS ONE.

[30] Xie L., Xia K., Xu X., Zhu M., Li H., Wang J., Chen M. (2025). Mortality burden and epidemiology of procedure- and device-related healthcare-associated infections in the United States, 1999–2023: A CDC WONDER analysis. Front. Public. Health.

[31] Slimings C., Riley T.V. (2021). Antibiotics and healthcare facility-associated Clostridioides difficile infection: Systematic review and meta-analysis—2020 update. J. Antimicrob. Chemother..

[32] Di Bella S., Sanson G., Monticelli J., Zerbato V., Principe L., Giuffrè M., Pipitone G., Luzzati R. (2024). Clostridioides difficile infection: History, epidemiology, risk factors, prevention, clinical manifestations, treatment, and future options. Clin. Microbiol. Rev..

[33] Viprey V.F., Clark E., Davies K.A. (2024). Diagnosis of Clostridioides difficile infection and impact of testing. J. Med. Microbiol..

[34] European Centre for Disease Prevention and Control (2024). Point Prevalence Survey of Healthcare-Associated Infections and Antimicrobial Use in European Acute Care Hospitals, 2022–2023.

